# Partially dissociative role of the left inferior frontal gyrus and left dorsolateral prefrontal cortex in reasoning

**DOI:** 10.1371/journal.pone.0312919

**Published:** 2024-12-02

**Authors:** Shane Fresnoza, Kjell Büsche, Maximilian Kern, Monica Christova, Sascha Freigang, Jochen A. Mosbacher, Roland H. Grabner, Anja Ischebeck

**Affiliations:** 1 Department of Psychology, University of Graz, Graz, Austria; 2 BioTechMed, Graz, Austria; 3 Otto Loewi Research Center, Physiology Section, Medical University of Graz, Graz, Austria; 4 Institute of Physiotherapy, University of Applied Sciences FH-Joanneum, Graz, Austria; 5 Department of Neurosurgery, Medical University of Graz, Graz, Austria; Institute for Research in Fundamental Sciences, ISLAMIC REPUBLIC OF IRAN

## Abstract

Reasoning is the ability to formulate inferences or conclusions from available information. The two major types, deductive and inductive, are thought to rely on distinct cognitive mechanisms and recruit separate brain areas. Neuroimaging studies yield mixed results; some found the left inferior frontal gyrus (IFG) activations for deductive reasoning and the left dorsolateral prefrontal cortex (DLPFC) for inductive reasoning. This assumption was put to the test in the present study. In two double-blinded, sham-controlled experiments, high-definition transcranial direct current stimulation (HD-tDCS) was used to systematically explore the left IFG’s and DLPFC’s causal role in deductive and inductive reasoning. Participants with no formal training in logic judged deductive and inductive arguments before and after 10 minutes of anodal, cathodal, or sham tDCS of the left IFG (Experiment 1, n = 20) or left DLPFC (Experiment 2, n = 21). Left IFG anodal tDCS impairs reaction times (RTs) for easy categorical (*p* = < .001) and propositional (*p* = .025) deductive arguments and the accuracy for easy inductive propositional arguments (*p* = .003). Meanwhile, regardless of the active stimulation conditions, left DLPFC tDCS shortens RTs (anodal: *p* = < .001, cathodal: *p* = .014) and increases accuracy (anodal: *p* = .029, cathodal: *p* = .001) for difficult categorical inductive arguments, but decreases accuracy (anodal: *p* = .027, cathodal: *p* = < .001) for difficult propositional inductive arguments. The overall results showed a partial dissociation of the left frontal lobe areas subserving the two types of reasoning and argument difficulty-dependent stimulation effects. This study extends knowledge of the neural basis of reasoning and hopefully inspires interventions that could augment reasoning impairments associated with normal aging and brain lesions.

## Introduction

Reasoning is ubiquitous in daily life and lies at the core of human intelligence [1,2]. Logical judgments are effortlessly made through goal-directed reasoning in simple situations, such as concluding that other caffeine-containing drinks are also stimulants because of previous experience with coffee. Conclusions are also drawn in occupational contexts, such as legal or medical decision-making, by integrating and interpreting bodies of interrelated evidence [3]. However, three main factors limit human’s capacity to understand reasoning fully. First, reasoning studies often use artificial materials or tasks that do not reflect the processes people typically engage in when they reason in everyday life [1,4]. Second, there is no unified cognitive theory on which to model reasoning [5]. And third, not much is known about the neuroanatomical and functional correlates of reasoning. The present study will contribute to this latter point by investigating the causal role of the left inferior frontal gyrus (IFG) and left dorsolateral prefrontal cortex (DLPFC) in deductive and inductive reasoning. Elucidating the neural mechanism will provide an important step in further understanding the neuroanatomical underpinnings of reasoning. Most importantly, from a clinical perspective, the knowledge we aimed to gain could help design new interventions to augment reasoning performance in healthy elderly and neuropsychiatric conditions like schizophrenia [6,7]. Older adults often have difficulties inhibiting currently-held beliefs (belief bias), resulting in reduced logically correct decisions [8]. On the other hand, in schizophrenia, one model of delusion-formation posits that delusional thought may be the result of impaired reasoning since individuals with a greater degree of delusional ideation exhibit more reasoning biases during tasks of hypothesis testing and probability judgments [9]. Patients with delusions often exhibit “jump-to-conclusions” reasoning bias by making decisions with minimal available information [10]. There is emerging evidence that noninvasive brain stimulation (NIBS) techniques such as transcranial direct current stimulation (tDCS) may decrease the rate of cognitive deterioration in older adults with and without mild cognitive impairment [11], as well as in patients with Alzheimer’s Disease or in pre-stages [12], and individuals with mood and schizophrenia-spectrum disorders [13]. Therefore, the potential of tDCS in enhancing the brain’s capacity to carry out complex cognitive functions such as reasoning is promising.

In recent years, investigation of the two main types of reasoning, deductive and inductive, using sentential, figural, and numerical stimuli, has been gaining ground. For sentential reasoning, the stimuli are verbal arguments or syllogisms containing two or more premises and a conclusion. In deductive reasoning (e.g., *“All humans are mortal*. *Socrates is human*. *Hence*, *Socrates is mortal”*), participants evaluate the validity (valid or invalid) of the conclusion depending on the information the premises provide [14,15]. In contrast, in inductive reasoning (e.g., *“Cats purr*. *A lion is a cat*. *Therefore*, *lions purr*.*”*), participants evaluate the plausibility or strength (weak or strong) of the conclusion depending not only on the information the premises provide but also on their background knowledge [14,16,17]. One open question in reasoning research is whether deduction and induction are simply two different kinds of the same reasoning process (in terms of the structure and/or content of the problems) or whether they are genuinely two different kinds of reasoning with different cognitive processes involved [16]. According to single-process theories such as the Mental Model Theory, formulating inductive and deductive inferences requires a single nonverbal process of constructing small-scale models or visuospatial representations of the problem’s premises [2,18–20]. Another single-process theory, the formal rule approach, suggests that reasoning requires retrieving and applying formal inferential rules for manipulating propositions to reach a conclusion [20,21]. In contrast, dual-process accounts assume that two separate systems rather than a single parsimonious mechanism underly reasoning. These two interactive systems (one fast, one slow) have complementary functions, and induction and deduction recruit them, albeit at different degrees [22,23]. In this framework, deduction relies more on controlled, slower, analytical, logic-based processing, which is working memory-dependent and of limited capacity (System 2). This makes reasoning more deliberate and typically more accurate [16,22–24]. In contrast, induction is mainly driven by processes (System 1) that are automatic and rapid, reliant on implicitly acquired world knowledge, unconscious, and without requiring controlled attention. These processes are thought to be working memory-independent and capable of processing large amounts of information [16,22–24].

Concerning the neural correlates of deductive and inductive reasoning, most evidence comes from neuroimaging studies contrasting task-related brain activations. In a positron emission tomography (PET) imaging study, deduction elicits left IFG activity, while induction recruits a large area, including the left medial frontal gyrus (MeFG), the left cingulate gyrus, and the left superior frontal gyrus [25]. In a follow-up functional magnetic resonance imaging (fMRI) experiment, there was stronger left IFG activation for deduction than in induction and a stronger left DLPFC activation for induction than in deduction [14]. In another PET study, inductive inference activates mostly left brain areas, including the superior and medial prefrontal cortices, while deductive inference primarily recruits right hemispheric area homologues of the language processing regions (Wernicke’s area and Broca’s area) in the left hemisphere [26].

The recruitment of the left IFG for deductive reasoning is consistent with greater working memory requirements, particularly involving the phonological loop, than inductive reasoning [14]. Deduction also requires greater syntactic processing because logic relies on the syntax that determines the validity of deductive arguments [14]. A quantitative meta-analysis of neuroimaging studies also found that deductive reasoning recruits areas in the left hemisphere but that it depends on the argument form: categorical, propositional, or relational. Categorical deductive arguments (e.g., *“All As are Bs*. *All Bs are Cs*. *Therefore*, *all As are Cs”*.) are shown to recruit the left IFG and left basal ganglia (BG) because of the linguistic/syntactic process involved in reasoning [20]. Propositional deductive arguments (e.g., *“If there is an A*, *then there is a B*. *There is an A*. *Therefore*, *there is a B*.*”*) were found to activate the left posterior parietal cortex (PPC), left precentral gyrus (PG), and MeFG because these areas were associated with non-syntactic verbal processing, maintenance of abstract rules in memory, and attentional and motor processes [20]. Relational deductive arguments (e.g., *“A is to the left of B*. *B is to the left of C*. *Therefore*, *A is to the left of C*.*”*) recruits the bilateral PPC and right middle frontal gyrus (MFG) possibly because of visuospatial processes [20]. It thus makes sense to distinguish between the different forms of deductive arguments. On the other hand, for inductive reasoning, recruitment of the left DLPFC might indicate an increased use of world knowledge in generating and evaluating hypotheses [14].

Neuroimaging results reveal some insights into reasoning, but they show merely regions of correlated activity; hence, causal relationships cannot be inferred [27,28]. Brain regions incidentally co-activated by a reasoning task are hard to separate from those critical to reasoning. Applying NIBS techniques can bypass this problem by facilitating or inhibiting neuronal activity in specific brain areas. TDCS and repetitive transcranial magnetic stimulation (rTMS) are the most commonly used neuromodulation interventions. TDCS delivers a low-intensity direct electrical current to the brain using surface electrodes. During stimulation, alterations of neuronal membrane potentials occur due to the opening or closing of voltage-gated ion channels [29]. In the motor cortex, tDCS has a polarity-dependent effect on neuronal excitability: anodal (positive current) stimulation brings the resting membrane potential closer to depolarization (increasing the likelihood of neuronal firing), increasing neuronal excitability. In contrast, cathodal (negative current) stimulation brings the resting membrane potential closer to hyperpolarization (which decreases the likelihood of neuronal firing), decreasing neuronal excitability [30]. The after-effects of excitatory and inhibitory brain stimulation paradigms are due to long-lasting synaptic efficacy changes such as long-term potentiation (LTP) and long-term depression (LTD), respectively [29–31].

The use of NIBS to explore reasoning is promising. However, there have been very few NIBS studies conducted on this topic, and they have addressed different research questions and targeted different brain areas. For example, 10 Hz rTMS of the primary visual cortex disrupts the construction of visual images and facilitates reasoning for deductive relational arguments [32]. In other studies, bilateral superior parietal lobule (SPL) 1 Hz rTMS impairs reasoning for abstract (e.g., “*No B are Z*. *All P are B*. *No P are Z*.*”*) and incongruent categorical deductive arguments (e.g., “*No pigeons are mammals*. *All pigeons are birds*. *No birds are mammals*.*”*) without affecting congruent deductive arguments (e.g., *“No mammals are birds*. *All dogs are mammals*. *No dogs are birds*.*”*) [33]. Similarly, right SPL 10 Hz rTMS disrupts reasoning for uncertain relational deductive arguments (e.g., *“The apple is to the left of the lemon*. *The lemon is to the left of the pear*. *The mango is to the left of the orange*. *The lemon is to the left of the mango*.*”*) where participants cannot give a certain answer because no matter what arrangement they imagine, they can always think of another possibility that is also consistent with the premises, like the order of the pear and the mango [34]. On the other hand, right PPC but not left DLPFC anodal tDCS and sham enhance performance in deductive and inductive indeterminate relational problems [35] These are problems with premises that convey indeterminate arrangements from which multiple models can be constructed. For example, Is the “Plate-Knife-Fork-Spoon-Bowl” positioning correct given the following premises: *“The plate is to the left of the knife*. *The fork is to the right of the knife*. *The spoon is to the left of the bowl*. *The spoon is to the right of the knife*.*”* These studies collectively support the important role of spatial processing in the parieto-occipital area for reasoning, as neuroimaging literature suggests [17,20]. Meanwhile, other studies performed anodal tDCS on the left DLPFC to explore the effect of emotion on analytical reasoning [36] and the right DLPFC to explore its causal role in numerical induction [37]. Right ventrolateral PFC continuous theta burst stimulation (cTBS) and bilateral IFG 1 Hz rTMS were also conducted to explore the impact of belief bias on reasoning [33,38,39]. So far, however, no study has yet explored the causal role of the left IFG and DLPFC for verbal deductive and inductive reasoning using NIBS, respectively.

The current study explores the dissociation of left frontal lobe areas, subserving deductive and inductive reasoning. In two separate experiments, anodal, cathodal, and sham stimulation were applied to the left IFG (Experiment 1) and left DLPFC (Experiment 2) using a high-definition tDCS (HD-tDCS) montage. Compared to conventional bipolar (two-electrode) tDCS with wide-spread electric field distribution, the HD-tDCS set-up constrains the spatial distribution of current radiating from the central electrode, ensuring higher stimulation focality [40,41]. Verbal deductive and inductive reasoning tasks with easy and difficult categorical and propositional arguments were performed before and after stimulation. Considering the preferential activations of the left IFG for deductive reasoning and left DLPFC for inductive reasoning [7,14,25,42], the hypothesis is that anodal and cathodal tDCS of the left IFG would influence deductive more than inductive reasoning, while anodal and cathodal tDCS of the left DLPFC would modulate inductive more than deductive reasoning. Furthermore, considering the findings of Prado et al. (2011) meta-analysis, an expectation is that there will be more robust effect of left IFG stimulation on the categorical than propositional arguments. As there are also reports of stimulation and cognitive load interactions, with tDCS facilitating the performance of more difficult than easy tasks [43–45], another expectation is a more robust stimulation effect on the difficult than on the easy arguments.

## Experiment 1: Stimulation of the left IFG

### Materials and methods

#### Participants

The participants were 20 healthy young adults (8 females, 12 males; age range: 20 to 28 years; mean age ± standard deviation (SD): 24.47 ± 2.14 years) enlisted from December 1, 2018, to June 28, 2019, using private contacts, social media, circular mails, and advertisement and in-class recruitment in the University of Graz. The sample size was sufficient to achieve a statistical power (1-β) of 95% at an alpha level of 0.05 and a large effect size (Cohen’s *f* = 0.40) for repeated measures (within-subject) design (G*Power 3.1.9.2) [46]. To keep the sample population homogenous, participants must be native German speakers, have normal or corrected-to-normal vision, and be right-handed, according to the Edinburgh Handedness Inventory [47]. Moreover, to avoid the influence of task familiarity and prior experience with tDCS on reasoning performance, undergraduate psychology student volunteers must be naïve about brain stimulation and have not yet undertaken any courses in reasoning. Meanwhile, to ensure safety, participants were excluded if any contraindications to tDCS were present, such as any history of chronic medical or neuropsychiatric disorders (e.g., depression, epilepsy, and stroke), learning disability, brain injuries, neurosurgical intervention, intake of medications, substance abuse, pregnancy, and metallic or electrical implants in the body or the head [48]. Participants were told to avoid alcoholic drinks at least 24 hours before the experiments. The University of Graz Ethics Committee approved the study (ethic number: 39/91/63), and all performed experimental procedures complied with the principles of the Helsinki Declaration regarding human experimentation. All participants gave written informed consent and were compensated for their participation with course credit points equivalent to the time they spent in the experiments.

#### Experimental design and procedure

In a double-blinded, randomized, sham-controlled design, each participant took part in three experimental sessions (two with active tDCS stimulation (anodal and cathodal) and one with sham stimulation). The experimental conditions were counterbalanced across subjects, and there was an inter-session interval of at least one week to avoid carry-over effects. To ensure double-blinding, neither the experimenter nor the participant knew the stimulation conditions. Another research team member set up the stimulation parameters, and the stimulator was kept out of sight during the stimulation. Before the experiment began, the participants were briefed about the purpose of the study and given detailed instructions. Subsequently, the left IFG was localized using the international 10–20 electroencephalogram (EEG) coordinates. Individual participants’ head dimensions were measured using a tape measure (in cm). The midpoints between the nasion and inion and between the right and left pre-auriculars were identified and marked on the scalp using a washable pencil marker. The intersection point was the vertex and was used to position the EEG cap on the participants’ heads. The EEG cap was mounted with the Cz electrode matching the vertex’s location. To accommodate various head sizes, three (size 54, 56, and 58) elastic EEG caps (EasyCap GmbH, Herrsching, Germany) were used to locate the position of the left IFG at the F7 electrode. After locating the left IFG, the EEG cap was removed and replaced with the customized elastic head cap that houses five plastic electrode holders (HDM-Biosemi Electrode Holder, Soterix Medical Inc., New York, USA). The central electrode holder was positioned over the location of the left IFG. Five sintered ring silver/silver chloride (Ag-AgCl) electrodes were inserted into the electrode holders after filling them with clear conductive gel. A plastic cover was placed on the electrode holders, and the electrodes were connected to the HD-tDCS adaptor. Before and after the stimulation, the participants evaluated different verbal deductive and inductive reasoning arguments. The total duration of the experimental session, including the preparation, lasted about 40 minutes.

#### HD-tDCS

HD-tDCS or sham stimulation was delivered using a 4 × 1 electrode montage. The current source was a battery-driven, direct-current stimulator (DC-STIMULATOR PLUS, NeuroConn Gmbh, Ilmenau, Germany) connected to a multichannel adaptor, which diverts the current to the HD-tES electrodes (4 × 1 HD-tES adaptor, Soterix Medical Inc., New York, NY, United States). The five sintered ring (Ag/AgCl) electrodes had a diameter of 1.2 cm and were housed in plastic electrode holders (base diameter: 24 mm; height: 13 mm) attached to a customized elastic head cap. The stimulating central electrode (anode, cathode, or sham) was centered over the left IFG (F7) according to the international 10–20 EEG system for electrode placement. The locations of the four return electrodes corresponded roughly to the F5, AF7, FT7, and F9 electrode positions. For active stimulation (anode and cathode), the current was 1.5 mA continuously for 10 minutes. The current density under the center electrode was 0.199 mA/cm^2^ calculated using the equation:

$$J=\frac{I}{2\times \pi \times r}$$

**Fig 1:**
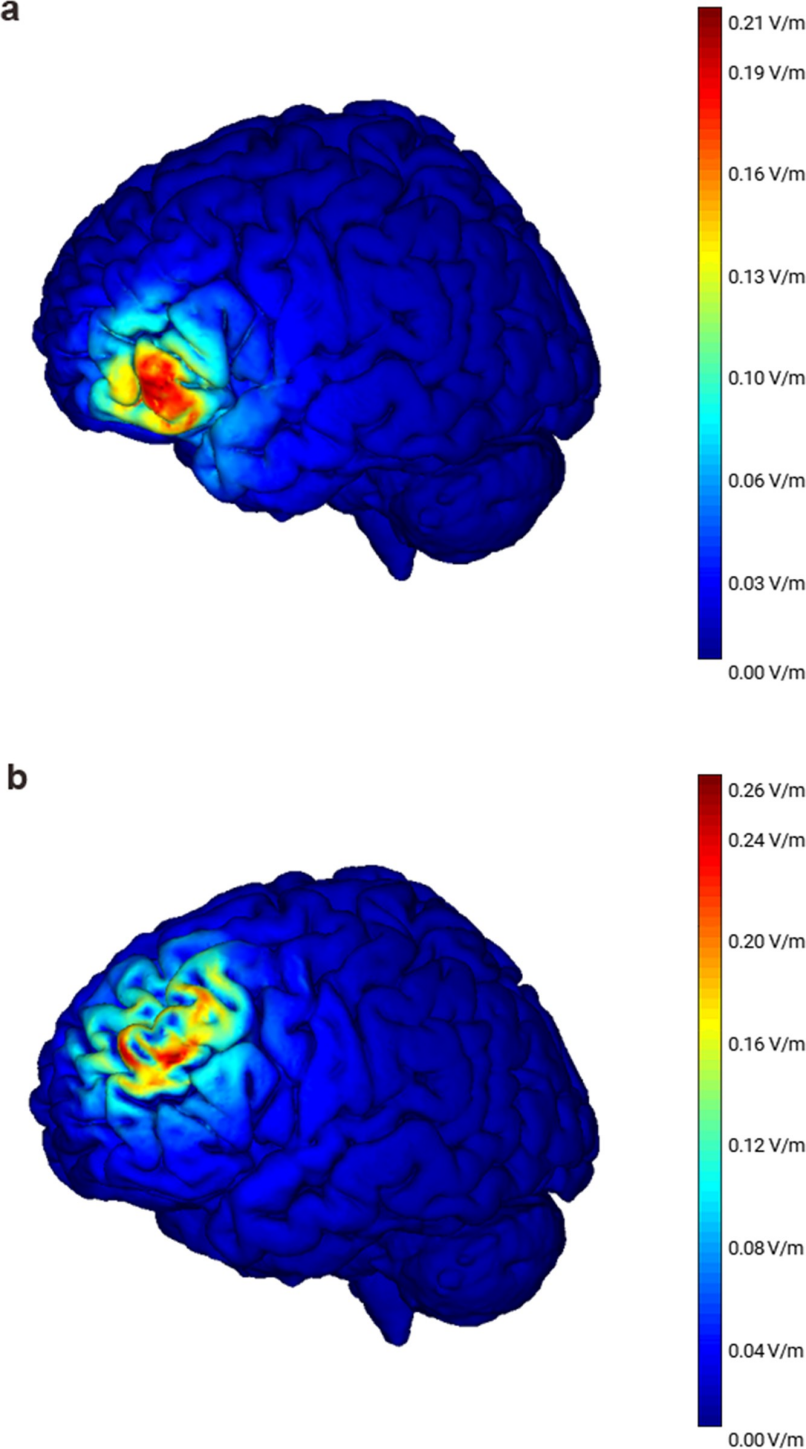
Simulated electric field map (E_n) on adult human brain’s gray matter surface induced by HD-tDCS. **(a)** Left inferior frontal gyrus stimulation in Experiment 1. The central electrode is at F7, and the four return electrodes are at F5, AF7, FT7, and F9 electrode positions (10–20 EEG System). Peak electric field strength of 0.21 V/m (red) at the pars opercularis and pars triangularis. **(b)** Left dorsolateral prefrontal cortex stimulation in Experiment 2. The central electrode is at F3, and the four return electrodes are at AF3, F1, F5, and FC3 electrode positions (10–20 EEG System). Peak electric field strength of 0.26 V/m (red) at Brodmann’s areas 9 and 46.

Where *J* is the current density, *I* represents the current intensity, π the Pi constant (3.14), and *r* is the electrode radius. The return current was equally divided among the four reference electrodes; therefore, the current density underneath them was 0.049 mA/cm^2^. Fig 1A shows the simulated electric field distribution in a realistic 3D head model using StimViewer software (StarStim, Neuroelectrics Barcelona, Spain) as described in previous studies [49,50]. At the beginning and end of stimulation, the current was slowly ramped up and down to 1.5 mA over 10 seconds, respectively. To minimize the tingling skin sensation, electrode resistance was kept below 2.5 quality units (impedance and voltage ratio). The current was applied for 30 seconds for sham stimulation and then turned off automatically. This procedure ensured that participants felt a similar skin sensation during sham stimulation, making it indistinguishable from the real stimulation condition. All stimulation parameters conformed to the safety guidelines for tDCS [51]. At the end of the experimental session, stimulation-related adverse symptoms were documented using a standard tDCS questionnaire [52]. In addition, the experimenter and participants were asked to identify what type of stimulation condition they had performed and received, respectively.

#### Stimuli and task

**Fig 2:**
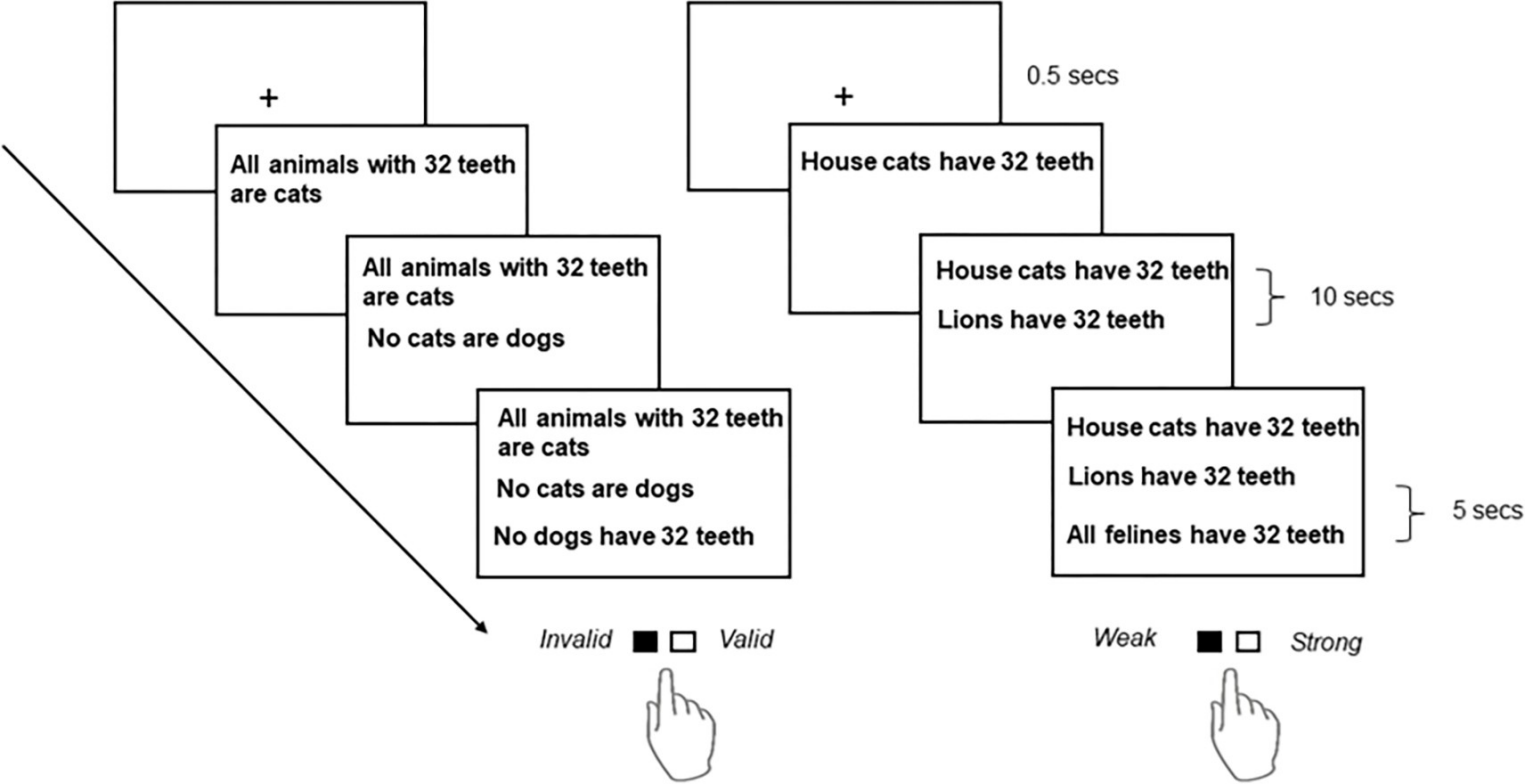
Reasoning task. A trial starts with the appearance of the fixation cross for 0.5 secs. The first premise appears after the fixation cross disappears, and the second premise appears 10 seconds after the first one. The conclusion is then shown 5 seconds after the second premise. For deductive reasoning (left), participants had to indicate whether the conclusion was valid (right button) or invalid (left button). For inductive reasoning (right), participants had to indicate whether the conclusion was strong (right button) or weak (left button). Task adapted from Goel and Dolan (2004) [14].

The task procedure and stimulus materials were created based on previous neuroimaging studies on reasoning [14]. The stimuli (in German) were verbal arguments or syllogisms with two premises and one conclusion. They were written in black letters (Arial 32 font) and presented against a white background (Fig 2). There were 240 different arguments divided into three sets (80 arguments per set), which were then randomized and counterbalanced in a Latin square design across stimulation conditions and participants. During the experiment, the participants were given blocks of 40 arguments (20 deductive and 20 inductive) before and after stimulation. The deductive arguments were divided into categorical (10) and propositional (10) forms. There were no relational deductive arguments because this form was more consistently associated with bilateral PPC and right MFG activations than propositional or categorical arguments [20]. Per form, the deductive arguments were equally divided by difficulty (5 easy and 5 difficult). However, this division made an equal distribution of valid and invalid conclusions impossible; hence, easy and difficult arguments were randomized such that there were two valid and three invalid answers or vice versa. The inductive arguments were formulated in a way that they heavily rely on prior knowledge, and were only in propositional form. There were 10 easy and 10 difficult arguments, each containing 5 with weak conclusions and 5 with strong conclusions, for a total of 20 arguments.

The logical validity of deductive arguments and the plausibility of inductive arguments were initially assessed by two scientific team members (experts, one female). On the other hand, the argument’s difficulty was assessed based on the reaction times collected from a pretest involving 4 undergraduate students (non-experts, two females) who did not participate in the main experiment. The median RT was determined separately for the deductive (2.91 seconds) and inductive (3.09 seconds) arguments. The cut-off time for both types of arguments was 3 seconds. Arguments with RTs below this threshold were classified as “easy,” and arguments with RTs above it were considered “difficult.” Moreover, the number of words in the premises and conclusion were matched, so the difficulty levels of deductive and inductive arguments are equal. On average, the number of words in a block for the easy (deductive: 16.33; inductive: 19.55) and difficult (deductive: 21; inductive: 25) arguments did not significantly differ (easy: *p* = .254; difficult: *p* = .228) between the two reasoning types. The deductive and inductive arguments were also matched based on content such that if there is a deductive argument about dogs and cats (e.g., All animals with 32 teeth are cats; cats are not dogs; dogs have not 32 teeth), there is also a corresponding inductive argument about them (e.g., House cats have 32 teeth; lions have 32 teeth; all felines have 32 teeth). Cautions are made to ensure that the arguments do not contain emotionally negative content because this interferes with reasoning [36].

The experiments took place in a quiet and well-lit room. The participants were seated in front of a 24-inch computer screen (refresh rate: 60 Hz; resolution: 1920 × 1080 pixels) at a distance of 50 cm. The experiment began with a screen display containing information about the order of arguments presentation (20 deductive-20 inductive or 20 inductive-20 deductive) and instructions on how to answer using the response box buttons (Cedrus Response Pad RB740, Cedrus Corporation, California, USA). Each trial began with a fixation cross in the middle of the screen for 0.5 seconds (Fig 2). Then, the first premise was shown in the upper third of the screen. After 10 seconds, the second premise appeared at the center of the screen. After 5 seconds, the conclusion appeared in the bottom third of the screen. Premises and conclusions remained on the screen until the participants pressed a button. The participants were instructed to carefully read each premise and make a judgement about the conclusion. Participants had to judge whether the conclusion was valid or invalid based only on the given premises for deductive arguments. On the other hand, for inductive arguments, participants were asked to judge the strength of the conclusion based on their world knowledge and the amount of doubt they had about it. In other words, participants were told to consider a conclusion “weak” or “strong” if it casts greater or less doubt, respectively. Participants were told to respond rapidly and accurately using their right index finger. The next trial began 2 seconds after a response. Reaction time (RT) and errors were recorded for each trial. RT was measured from the onset of the conclusion until a keypress. Meanwhile, the participants’ accuracy in deduction was evaluated according to logical reasoning, while their accuracy for induction was evaluated based on consistency with general knowledge [53,54]. A Psychopy-based program (Psychopy Software in Python, University of Nottingham, UK) was used for stimulus presentation and response recording [55].

#### Statistical analysis

*Reaction time*. The individual participant’s average RT for deductive and inductive arguments was calculated before and after stimulation. RTs beyond +2 standard deviations away from the mean (outliers) and RTs from incorrect trials were excluded. To prevent bias or incorrect removal of RTs (e.g., long RTs for difficult trials), outlier removal was conducted separately for easy and difficult trials based on their respective mean. Data distribution analysis (Shapiro-Wilk test) revealed a log-normal distribution (*p* = < .001), typical for positive values (skewness: 1.835; kurtosis: 4.218). Log-transformation improved the data skewness (.062) and kurtosis (-.477), but its distribution remained non-normal (*p* = < .001). Therefore, the raw RTs were analyzed using a generalized linear model (GLM). Unlike conventional approaches (e.g., general linear model), GLM is robust in modelling continuous, non-negative, and skewed data, as well as data with missing observations [56]. The RTs for easy and difficult trials were modelled separately because a single model may fail to reveal stimulation-specific effects (S1 Data). For instance, there will be comparable RTs when the stimulation increases RTs for easy trials without changing RTs for difficult trials. This is also possible for the reverse scenario: decreased RTs for difficult trials without a change in RTs for the easy trials.

The RTs were modelled using a Gamma probability distribution with an identity link function, particularly suitable for data containing only positive numbers. The separate models for easy and difficult trial RTs contained the “stimulation” condition (anodal, cathodal, and sham), “reasoning” type (deductive categorical, deductive propositional and inductive propositional), and “time” (before and after stimulation) as within-subject factors. Model fitting was assessed by comparing the Akaike information criterion (AIC) and deviances of the full and baseline or intercept-only models. A decrease or increase (>2) in the AIC difference upon independent factors addition signals an improvement or worsening of the fit, respectively [57]. The maximum likelihood estimation method was used for estimating the model’s parameters. Statistically significant effects (*p*-value < 0.05), as determined by Wald chi-square statistics, were further explored using Bonferroni corrected post hoc pairwise comparisons. Cohen’s *f^2^* was used as a measure of effect size (< 0.02 = negligible, > 0.02 = small, > 0.15 = medium, and > 0.35 = large) because it was appropriate for multiple regression models [58,59]. All values were expressed as mean ± standard error of the mean (SEM). The analyses were performed using SPSS version 27 software (IBM Corp., Armonk, NY, USA).

*Accuracy rate*. The AR or the percentage of correct responses (number of correct trials/total trials x 100) was computed separately for easy and difficult trials before and after stimulation (S1 Data). The distribution of ARs was also non-normal based on the Shapiro-Wilk test (*p* = < .001; skewness: -.669; kurtosis: -.322), and retesting log-transformed data did not solve this issue (*p* = < .001; skewness: -1.437; kurtosis 3.360). Therefore, the same statistical procedure used for the RTs was used to analyze the raw ARs. The ARs for easy and difficult trials were modelled separately using Poisson regression with a log-link function, an alternative regression model more appropriate for bounded values (e.g., rates, intensity, etc.), and outcome variables with low counts [60].

### Results

The participants tolerated the stimulations, and none reported symptoms requiring medical intervention (e.g., headaches, dizziness, or nausea). In all sessions, tolerable scalp itchiness was reported by all participants at the beginning of the stimulation. The proportion of participants and experimenter’s stimulation type correct guesses (S1 Table) were all below the chance level (50%), and the chi-square tests were not significant (participants: X^2^ (2, n = 60) = 1.19, *p* = .551; experimenter: X^2^ (2, n = 60) = 0.43, *p* = .803). The chi-square test results indicate that the answers (sham or active) were independent of the stimulation type they received or performed, confirming the reliability of the double-blinding.

#### Reaction time

Out of the total 4800 reaction times (RTs) recorded, 925 RTs (19.27%) from incorrect trials and 76 outlier RTs (1.38%: easy trials = 24, difficult trials = 52) were excluded from the analysis. The data’s remaining 79.35% (3809 RTs) were used for analysis. The easy trials full model had 1960 RTs, while the difficult trials full model had 1849 RTs. The full model’s goodness-of-fit, as indicated by AIC (easy trials = 7967.873; difficult trials = 8693.348) and Deviance/df (easy trials = .488; difficult trials = .505), was better than the baseline model’s AIC (easy trials = 8076.986; difficult trials = 8785.986) and Deviance/df (easy trials = .517; difficult trials = .694).

**Fig 3:**
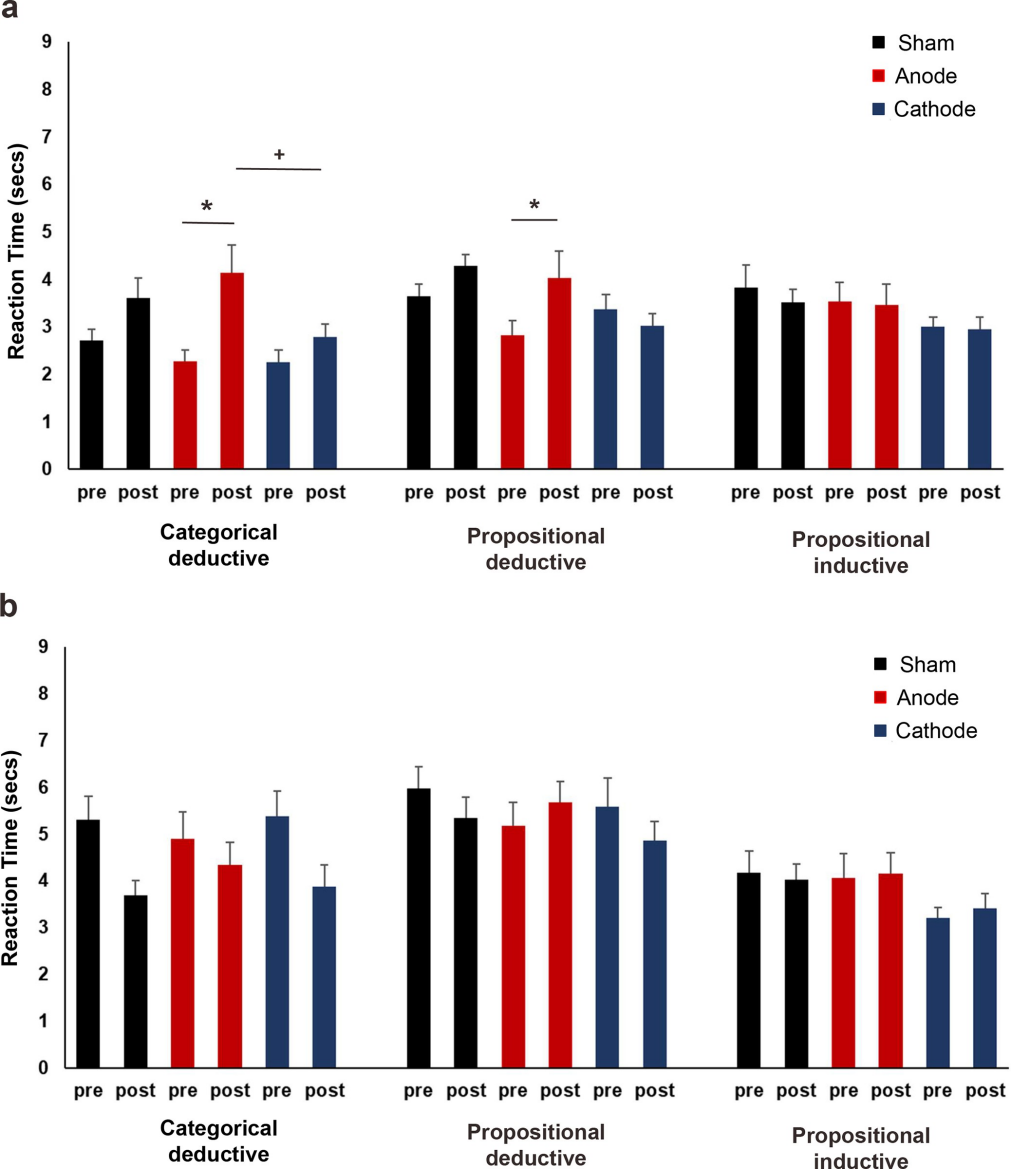
Reaction times for deductive (categorical and propositional) and inductive (propositional) arguments before and after left inferior frontal gyrus tDCS. **(a)** RTs for easy trials. Anodal stimulation significantly increased the RTs for categorical and propositional deductive arguments. The post-stimulation RTs were significantly longer for categorical deductive arguments after anodal than cathodal stimulation. **(b)** RTs for difficult trials. There were no significant stimulation-specific effects on RTs. pre = before stimulation, post = after stimulation, * = indicates significant differences before and after stimulation, + = indicates significant differences between stimulation conditions. Pairwise comparisons were Bonferroni corrected with a significance level of *p* ≤ 0.05. Data are presented as mean ± standard error of mean.

S2 Table shows the results of the GLM for raw RTs and ARs. For the easy trials RTs, the main effect of reasoning type was significant (χ^2^ = 26.89, df = 2, *p* = < .001, *f^2^* = .039). Participants were faster in judging categorical deductive arguments (2.89 sec ± .09 sec) than propositional deductive (3.55 sec ± .10 sec) and propositional inductive (3.37 sec ± .08 sec) arguments (*p*s = < .001). However, there was no significant difference in RTs for propositional deductive and inductive arguments (*p* = .445). The main effect of time was also significant (χ^2^ = 22.05, df = 1, *p* = < .001, *f^2^* = .041) as there was an overall slowing in judging arguments after stimulation. Post hoc tests of the significant reasoning and time interactions (χ^2^ = 26.54, df = 2, *p* = < .001, *f^2^* = .056) revealed that RTs slowing was only significant for categorical deductive arguments (*p* = < .001) as the overall pre- and post-stimulation RTs for propositional deductive (*p* = .134) and propositional inductive (*p* = .999) arguments were comparable. In addition, the main effect of the stimulation condition was significant (χ^2^ = 34.89, df = 2, *p* = < .001, *f^2^* = .005). Participants were generally slower in judging arguments in the cathodal (2.87 sec ± .08 sec) condition compared to sham (*p* = < .001) and anodal (*p* = .001) conditions, while their RTs in the sham (3.56 sec ± .10 sec) and anodal (3.38 sec ± .10 sec) conditions were comparable (*p* = .535). Furthermore, the stimulation and time interactions (χ^2^ = 15.76, df = 2, *p* = < .001, *f^2^* = .089) and the stimulation, reasoning, and time interactions (χ^2^ = 14.19, df = 4, *p* = .009, *f^2^* = .184) were significant. Post hoc tests for the three-way interactions revealed that participants became significantly slower in judging categorical (*p* = < .001) and propositional (*p* = .025) deductive arguments after anodal tDCS (Fig 3A). Additionally, their post-anodal stimulation RTs for categorical deductive arguments were significantly slower than their RTs after cathodal tDCS (*p* = .050).

In the difficult trials, the type of reasoning had a significant effect (χ^2^ = 78.31, df = 2, *p* = < .001, *f^2^* = .049) on RTs. Participants were faster in evaluating propositional inductive arguments (3.83 sec ± .09 sec) than categorical deductive (4.60 sec ± .14 sec) and propositional deductive (5.48 sec ± .17 sec) arguments (all *p*s = < .001). For the deductive arguments, participants were notably faster in evaluating categorical than propositional type (*p* = < .001). The effect of time was also significant (χ^2^ = 4.86, df = 1, *p* = .027, *f^2^* = .028), as participants generally became faster in evaluating difficult arguments after stimulation. However, the post hoc tests for the significant interactions of reasoning and time (χ^2^ = 12.73, df = 2, *p* = .002, *f^2^* = .092) revealed that participants were only significantly faster in evaluating categorical deductive (*p* = .006) but not propositional deductive (*p* = .999) and propositional inductive (*p* = .999) arguments (Fig 3B). The analysis did not reveal any significant effects of tDCS on the difficult trial’s RTs (S2 Table).

#### Accuracy rate

The full models for both easy and difficult trials are a good fit for the data. This is evident from their lower AIC (easy trials = 3099.070; difficult trials = 3451.231) and Deviance/df (easy trials = 2.352; difficult trials = 3.461) compared to the baseline model’s AIC (easy trials = 3369.271; difficult trials = 3719.687) and Deviance/df (easy trials = 3.088, difficult trials = 4.139).

**Fig 4:**
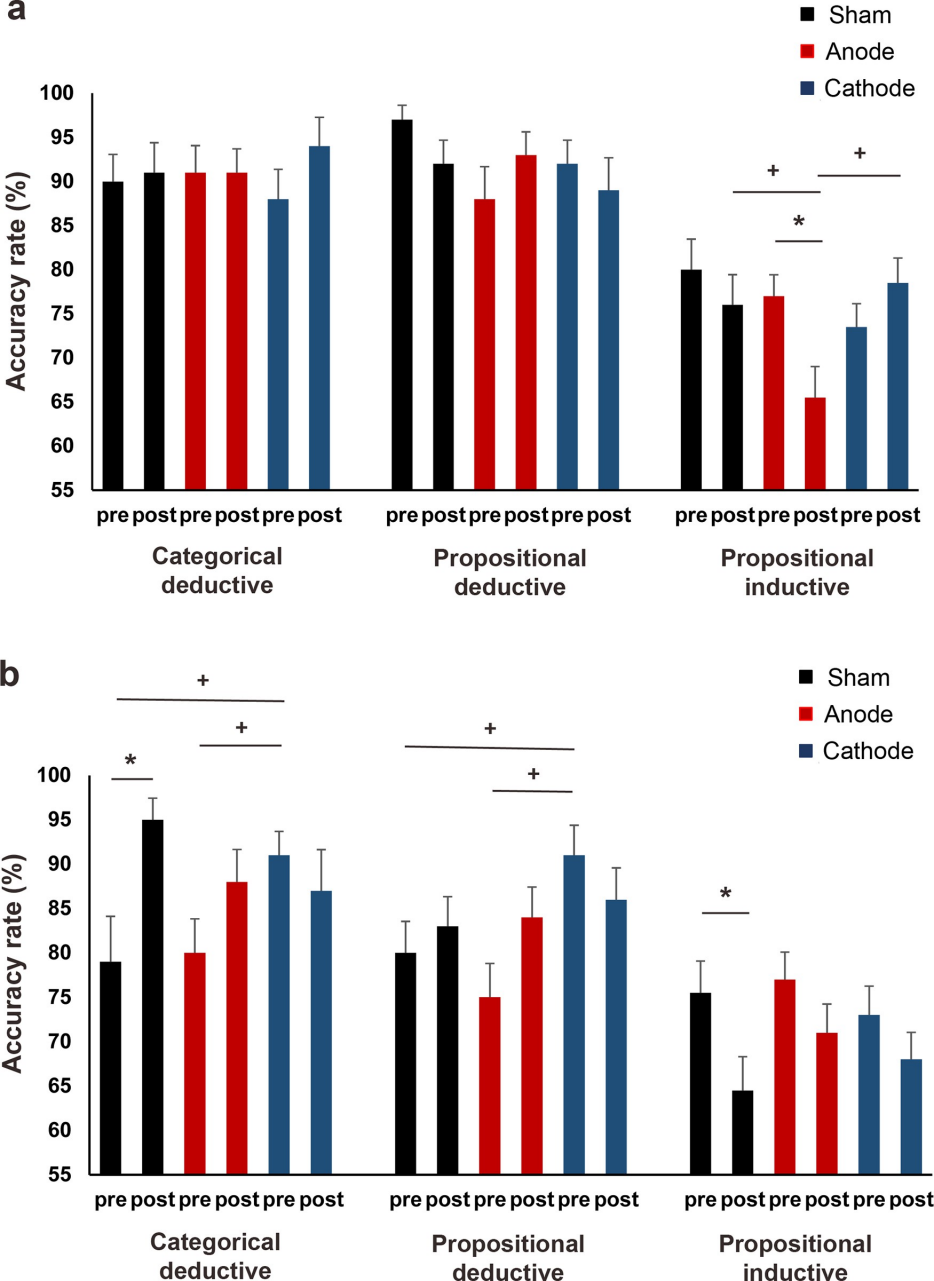
Accuracy rates for deductive (categorical and propositional) and inductive (propositional) arguments before and after left inferior frontal gyrus tDCS. **(a)** ARs for easy trials. Anodal stimulation significantly reduced the ARs for propositional inductive arguments. The post-anodal stimulation ARs for propositional inductive arguments were significantly lower than after sham and cathodal stimulations. **(b)** ARs for difficult trials. ARs significantly increased and decreased for categorical deductive and propositional inductive arguments after sham stimulation, respectively. For categorical and propositional deductive arguments, the pre-stimulation ARs in the cathodal condition were significantly higher than in sham and anodal conditions. pre = before stimulation, post = after stimulation, * = indicates significant differences before and after stimulation, + = indicates significant differences in ARs between stimulation conditions. Pairwise comparisons were Bonferroni corrected with a significance level of *p* ≤ 0.05. Data are presented as mean ± standard error of mean.

For the ARs on easy trials, the main effect of reasoning type was significant (χ^2^ = 249.34, df = 2, *p* = < .001, *f^2^* = .581). The participant’s accuracy was comparable for judging categorical (90.82% ± .87%) and propositional deductive (91.79% ± .88%) arguments (*p* = .999), and both were higher (*p*s = < .001) than their accuracy for propositional inductive arguments (79.93% ± .79%). However, post hoc tests for the significant reasoning and time interactions (χ^2^ = 6.88, df = 2, *p* = .032, *f^2^* = .620) showed no significant difference between the pre- and post-stimulation accuracies for all argument types (all *p*s >.050). The main effect of the stimulation condition was also significant (χ^2^ = 9.75, df = 2, *p* = .008, *f^2^* = .182). While the participant’s overall accuracy in the anodal (83.62% ± .84%) and cathodal (85.50% ± .85%) conditions (*p* = .339) were comparable, only the former was significantly lower than the accuracy in the sham (87.36% *+* .86%) condition (*p* = .005). The stimulation and reasoning interactions (χ^2^ = 9.88, df = 4, *p* = .042, *f^2^* = .661), the stimulation and time interactions (χ^2^ = 7.46, df = 2, *p* = .024, *f^2^* = .006), as well as the stimulation, reasoning, and time interactions (χ^2^ = 21.29, df = 4, *p* = < .001, *f^2^* = .701) were all significant. The post hoc tests for the three-way interactions showed that the participant’s accuracy for propositional inductive arguments significantly decreased after anodal tDCS (*p* = .003), and this post-anodal stimulation accuracy reduction was significantly larger than those after sham (*p* = .012) and cathodal (*p* = < .001) tDCS (Fig 4A).

The GLM findings for the difficult trials were similar to the easy trials’ RTs (S2 Table). The main effect of reasoning type was significant (χ^2^ = 186.42, df = 2, *p* = < .001, *f^2^* = .421). Participants were significantly more accurate in judging categorical (86.48% ± .85%) and propositional deductive (83.02% ± .83%) arguments than propositional inductive (71.37% ± .77%) arguments (*p*s = < .001). Additionally, for deductive arguments, participants were more accurate at judging categorical than propositional type (*p* = .011). The interaction between reasoning and time was also significant (χ^2^ = 40.54, df = 2, *p* = < .001, *f^2^* = .445). Post-hoc tests revealed that accuracy significantly increased for categorical deductive (*p* = .001) and decreased for propositional inductive (*p* = < .001) arguments after stimulation. Moreover, stimulation had a significant main effect (χ^2^ = 10.09, df = 2, *p* = .006, *f^2^* = .012). Participants were equally accurate in the sham (78.98% ± .81%) and anodal (78.97% ± .81%) conditions (p = .999), but both were significantly lower (sham: *p* = .019; anode: *p* = .018) than their accuracy in the cathodal condition (82.16% ± .83%). The stimulation and reasoning interactions (χ^2^ = 21.37, df = 4, *p* = < .001, *f^2^* = .535), stimulation and time interactions (χ^2^ = 13.41, df = 2, *p* = .001, *f^2^* = .031) and stimulation, reasoning and time interactions (χ^2^ = 22.57, df = 4, *p* = < .001, *f^2^* = .560) were also significant. The post-hoc tests for the three-way interactions revealed significant differences in baseline accuracies between stimulation conditions. Baseline accuracy was higher before cathodal tDCS than sham (*p* = .006) and anodal (*p* = .026) tDCS for categorical deductive arguments. It was also higher than sham (*p* = .026) and anodal (*p* = < .001) tDCS for propositional deductive arguments (Fig 4B). No significant differences in baseline accuracies existed between stimulation conditions for propositional inductive arguments. Interestingly, sham stimulation increased participants’ accuracy for categorical deductive arguments (*p* = < .001) and decreased their accuracy for propositional inductive arguments (*p* = .005) (Fig 4B).

#### Summary of tDCS-specific effects

When the left IFG was stimulated with anodal tDCS, the participant’s response time slowed significantly for easy categorical and propositional deductive arguments. It also reduced the accuracy for easy propositional inductive arguments. On the other hand, active tDCS did not significantly impact the participant’s response time or accuracy for difficult arguments, regardless of the type of reasoning and argument.

### Discussion of Experiment 1

The first experiment explored the causal contribution of the left IFG for deductive and inductive reasoning using HD-tDCS. The results showed that the effects of tDCS were specific to easy arguments. There was a reduction in response times for deductive (categorical and propositional) arguments and a reduction in accuracy for inductive (propositional) arguments following anodal stimulation. These findings indicate that the left IFG plays a causal role in both reasoning types.

The initial hypothesis that left IFG anodal tDCS would have a stronger stimulation effect on deductive reasoning was contradicted by the results, as it had detrimental effects on both deductive and inductive reasoning. However, the present findings support previous work by Goel and colleagues, demonstrating left IFG activations for both types of reasoning, albeit with a greater activation seen in deduction [14,25]. Interestingly, for deductive reasoning, the interference (RT slowing) for both categorical and propositional arguments argues against the findings of a previous meta-analysis showing left IFG and BG recruitment only with categorical arguments [20] In addition, the present results did not align with observations of impaired syllogistic categorical deductive reasoning by left IFG rTMS [33,38]. For inductive reasoning, anodal tDCS had a negative effect on the accuracy for propositional arguments, which supports previous PET findings by Osherson and colleagues that found strong induction-related activation in left inferior frontal areas [26,61]. Anodal tDCS may impair both types of reasoning by increasing glutamatergic-dependent neuronal firing and reducing inhibitory GABA concentration, with the latter leading to active disinhibition of a cortical region [62–65]. These events can decrease the signal-to-noise ratio and interfere with rule-governed syntax processing, a known function of the left IFG’s pars triangularis area in language [14,20,66,67].

One argument against the proposed model is that anodal tDCS may have a stronger effect on deductive reasoning due to its higher engagement of syntactic processing and greater working memory requirements than inductive reasoning [14,68]. However, the findings suggest otherwise, as RT slowing for categorical deductive arguments only significantly differed from that after cathodal tDCS, while the accuracy reduction for propositional inductive arguments significantly differed from those after sham and cathodal stimulations. The interference in inductive arguments after IFG stimulation is more challenging to explain because their premises may present as a string of facts, making processing their structure less essential than in deduction [66]. Inductive inferences do not also have to be as dependent on language as deductive inferences because prior world knowledge (on which induction also depends) can be learned through observation [66]. However, this reliance on prior knowledge in induction can be costly, particularly in the presence of interference, as participants would require a larger working memory capacity to hold the information extracted from the premises and retrieve relevant world knowledge.

TDCS stimulation did not specifically affect difficult arguments (Figs 3B and 4B), which was surprising. Remote brain regions may be involved in processing these arguments that were unaffected by the HD-tDCS stimulation protocol used here. An example is the left supramarginal gyrus, which is known for its involvement in the phonological loop and understanding complex sentences [69–71]. Additionally, strong functional connections between the rostrolateral PFC and the inferior parietal lobule have been found in young adults during reasoning [72,73], which is similar to the "multiple demand system" in non-human primates that are thought to support reasoning ability during complex tasks [74]. On the other hand, the effects of stimulation on easy arguments could have been caused by a spillover effect on neighboring cortical areas, such as the left DLPFC [75]. A recent study has shown that left DLPFC anodal tDCS can increase perceived self-ability but not cathodal tDCS [45]. The authors argue that high perceived self-ability keeps individuals engaged in difficult tasks, but not simple ones, as they tend to disengage once they understand that effort is unnecessary. After anodal tDCS, which might have increased self-ability, participants may have spent less time exploring the relationship between premises and conclusions, especially when they assumed that the presented scenario was easy. As a result, participants may be more likely to make errors in judging conclusions, as seen in the decrease in accuracy for simple arguments after anodal tDCS.

In summary, Experiment 1 demonstrated that the left IFG supports deductive and inductive reasoning. Nevertheless, the findings are limited with regard to their support of the separation of deduction and induction.

## Experiment 2: Stimulation of the left DLPFC

### Materials and methods

#### Participants

Twenty-one healthy young participants (11 females and 10 males; age range: 21 to 31 years; mean age ± SD: 26.09 ± 2.75 years) were recruited from July 22, 2020 to August 25, 2021 for Experiment 2 following the exclusion and inclusion criteria in the first experiment. There were 17 undergraduate psychology students from the University of Graz and five high school graduates who were respondents to the online ads. None of them had undertaken any courses in reasoning, and none participated in Experiment 1. All were native German speakers and had normal or corrected to normal vision. Participants provided written informed consent before the start of the experiment and were compensated for their participation with course credit points equivalent to the time they spent in the study. COVID-19 prevention measures were practiced in Experiment 2.

#### Experimental design and procedure

The experimental design and procedures for Experiment 2 are identical to Experiment 1. After completing the pre-stimulation adverse events questionnaire, the individual participant’s left DLPFC was localized using an EEG cap (EasyCap GmbH, Herrsching, Germany) according to the international 10–20 system of electrode placing. Subsequently, participants evaluated different deductive and inductive reasoning arguments before and after tDCS stimulation. Participants completed the post-stimulation adverse effect questionnaire at the end of each experimental session. To assess whether the blinding was compromised, the participants and experimenter were asked to indicate whether they received or applied an active or sham stimulation, respectively. Experiment 2 also lasted for 40 minutes.

#### HD-tDCS

The same stimulator (DC-STIMULATOR PLUS, NeuroConn Gmbh, Ilmenau, Germany) and multichannel adaptor (4 × 1 HD-tES adaptor, Soterix Medical Inc., New York, NY, United States) were used in Experiment 2. Stimulation parameters were identical to Experiment 1 except for the electrode montage: stimulating/central electrode at the F3 electrode location corresponding to the left DLPFC, and the four return electrodes at the AF3, F1, F5, and FC3 electrode positions (10‐20 International EEG System). The electric field distribution simulation in a realistic 3D head model for left DLPFC stimulation is shown in Fig 1B (StarStim, Neuroelectrics Barcelona, Spain). All participants tolerated the HD‐tDCS well, and aside from the itching or tingling sensation at the beginning of stimulation, none reported other adverse effects.

#### Stimuli and task

Experiment 2 used the same stimulus materials as in Experiment 1. However, half of the propositional inductive arguments of Experiment 1 were replaced with a categorical form. There were 240 arguments divided equally over the three experimental sessions (80 arguments per session). Each session has a block of 40 arguments (20 deductive and 20 inductive) before and after stimulation. Each reasoning type has an equal number of categorial (10) and propositional (10) arguments with five easy and five difficult arguments for each argument form. Equal distribution of valid and invalid deductive conclusions, as well as weak and strong inductive conclusions, was not possible. Therefore, arguments were randomized such that there were easy and difficult deductive arguments with two valid and three invalid answers (or vice versa). Similarly, there were easy and difficult inductive arguments with two weak and three strong answers (or vice versa). Participants in Experiment 2 were also instructed to judge whether the conclusion was valid or invalid based only on the given premises for deductive arguments and to judge the strength (“weak” or “strong”) of the conclusion based on their world knowledge and the amount of doubt they had about the conclusion for inductive arguments. Reaction time (RT) and errors were recorded for each trial. No feedback was given about the correctness of the answer.

#### Statistical analysis

The statistical software and procedures for the second experiment were identical to that in Experiment 1. The raw RTs (Gamma probability distribution with identity link function) and ARs (Poisson regression with a log link function) were modeled with GLM, and significant results were explored by Bonferroni corrected post hoc tests (S1 Data). In the separate models for easy and difficult trials, the “stimulation” condition (anodal, cathodal, and sham), “reasoning” type (deductive and inductive), “argument” form (propositional and categorical), and “time” (before and after stimulation) were included as within-subject factors. All values are expressed as mean ± standard error of the mean (SEM).

### Results

In the second experiment, the proportion of participants and experimenter’s stimulation type correct guesses (S1 Table) were all below the chance level (50%), except for the experimenter’s guesses for the sham (52.63%). Nonetheless, the chi-square tests revealed no significant association between the answers and the actual stimulation conditions received by the participants (X^2^ (2, n = 56) = 2.75, *p* = .313) or performed by the experimenter (X^2^ (2, n = 56) = 5.58, *p* = .140). In other words, the participants and the experimenter could not accurately guess the actual stimulation type, indicating that they remained unaware of the stimulation conditions.

*Reaction time*. In Experiment 2, 4480 RTs were collected, slightly less than the 4800 RTs collected in Experiment 1. Due to COVID-19-related reasons, one participant only had an anodal session, two had sham sessions, and one had anodal and cathodal sessions in Experiment 2. However, their RTs were still included in the analysis because the generalized linear model (GLM) is robust in modeling data with missing observations [56]. Out of the full data set, 1120 RTs (25%) from incorrect trials and 84 outlier RTs (1.88%) were excluded (27 RTs from easy trials and 57 RTs from difficult trials). The RT distribution was log-normal (*p* = < .001) according to the Shapiro-Wilk test (skewness = 2.881; kurtosis = 15.14). There were improvements in the skewness (.271) and kurtosis (-.295) after log-transformation, but the distribution remained non-normal (*p* = < .001). The final GLM analysis was performed on the raw data’s 73.15% (3277 RTs). The full model’s goodness-of-fit to the data was indicated by their lower AIC (easy trials = 7062.901; difficult trials = 7732.473) and Deviance/df (easy trials = .426; difficult trials = .461) compared to the baseline model’s AIC (easy trials = 7168.801; difficult trials = 7805.479) and Deviance/df (easy trials = .457; difficult trials = .487).

**Fig 5:**
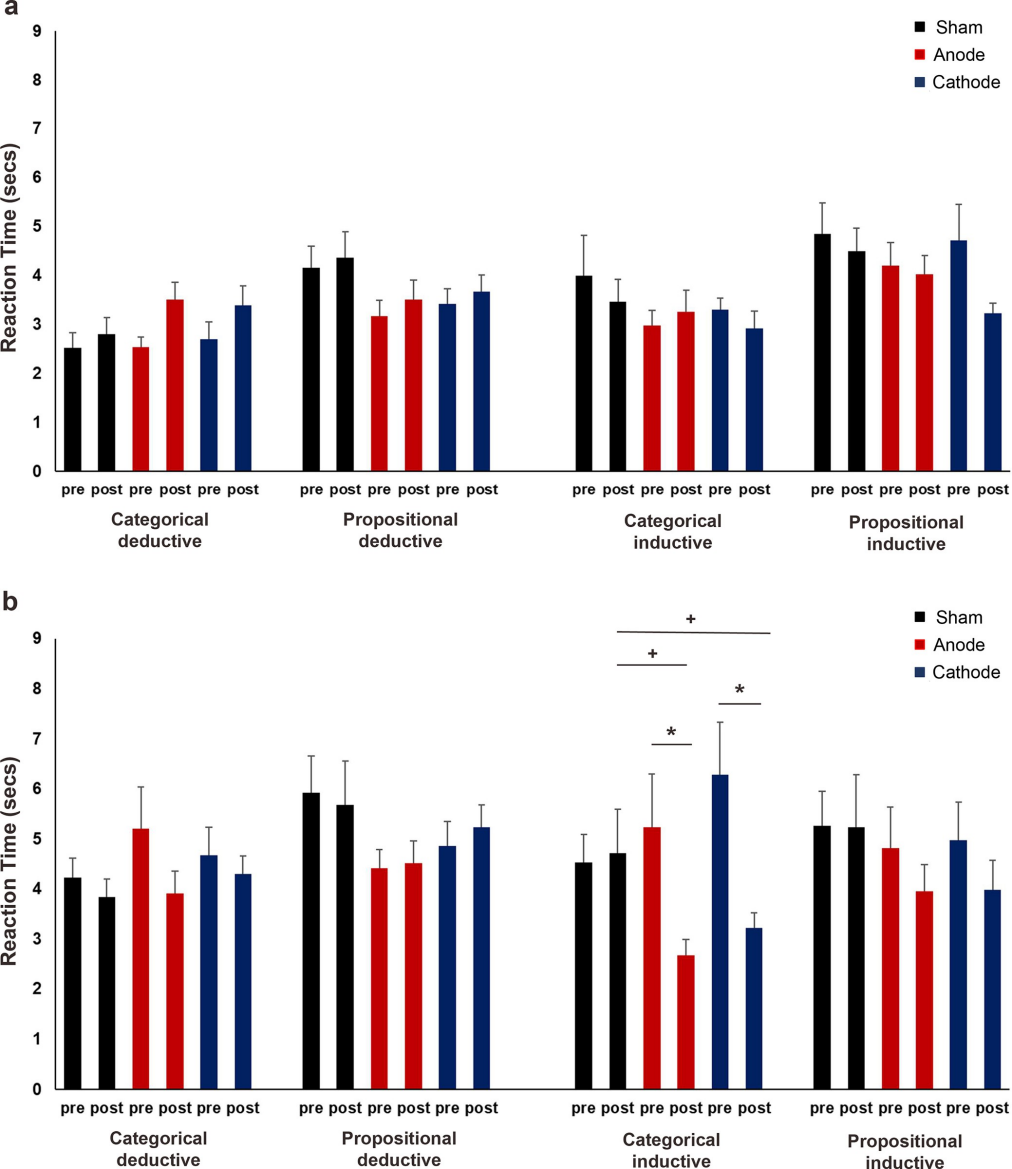
Reaction times for deductive and inductive arguments before and after tDCS stimulation of the left dorsolateral prefrontal cortex. **(a)** RTs for easy categorical and propositional deductive and inductive arguments. **(b)** RTs for difficult categorical and propositional deductive and inductive arguments. RTs for categorical inductive arguments significantly decreased after anodal and cathodal stimulations. The post-stimulation RTs for categorical inductive arguments were shorter in anodal and cathodal conditions than in sham. pre = before stimulation, post = after stimulation, * = indicates significant differences before and after stimulation, + = indicates significant differences in RTs between stimulation conditions. Pairwise comparisons were Bonferroni corrected with a significance level of *p* < 0.05. Data are presented as mean ± standard error of mean.

In the analysis of the easy trials (S3 Table), it was found that reasoning type and argument form had significant main effects on RTs (χ^2^ = 13.95, df = 1, *p* = < .001, *f^2^* = .106, and χ^2^ = 67.72, df = 1, *p* = < .001, *f^2^* = .035, respectively). Participants were faster to respond when presented with deductive arguments (3.31 sec ± .07 sec) than with inductive arguments (3.72 sec ± .09 sec). The same was true for categorical arguments (3.06 sec ± .07 sec) compared to propositional arguments (3.97 sec ± .09 sec). There was also a significant reasoning and time interaction (χ^2^ = 10.96, df = 1, *p* = < .001, *f^2^* = .023), with participants becoming slower to judge deductive arguments (*p* = .020) after stimulation. The stimulation condition’s main effect was also significant (χ^2^ = 11.54, df = 2, *p* = .003, *f^2^* = .081). Participants’ responses in the anodal (3.35 sec ± .09 sec) and cathodal (3.41 sec ± .09 sec) conditions were comparable (*p* = .999) and were both significantly faster than in the sham (3.79 sec *+* .10 sec) condition (anode: *p* = .004, cathode: *p* = .018). A significant interaction was also found between stimulation, reasoning, and argument (χ^2^ = 6.48, df = 2, *p* = .039, *f^2^* = .212). Post hoc tests revealed that participants were significantly faster in the anodal condition (*p* = .020) than in the sham condition in judging propositional deductive arguments (Fig 5A).

The analysis of the difficult trial’s RTs (S3 Table) showed that argument form had a significant impact on the participants’ response speed (χ^2^ = 6.58, df = 1, *p* = .010, *f^2^* = .012). RTs were faster for categorical arguments (4.41 sec ± .10 sec) than for propositional arguments (4.81 sec ± .11 sec). The main effect of time was also significant (χ^2^ = 18.45, df = 1, *p* = < .001, *f^2^* = .017), with participants responding faster overall after the stimulation. However, the significant interactions between reasoning and time (χ^2^ = 4.60, df = 1, *p* = .032, *f^2^* = .046) were only observed for inductive arguments (with shortened overall RTs, *p* = < .001), not deductive arguments (*p* = .653). Additionally, the argument and time interaction was significant (χ^2^ = 4.60, df = 1, *p* = .032, *f^2^* = .097), with response times shortening significantly for categorical (*p* = < .001), but not propositional arguments (*p* = .999). The stimulation condition also had a significant main effect (χ^2^ = 21.86, df = 2, *p* = < .001, *f^2^* = .065), with participants responding significantly faster in the anodal condition (4.13 sec ± .13 sec) than in the sham (4.92 sec ± .14 sec; *p* = < .001) and cathodal conditions (4.79 sec ± .14 sec; *p* = .001). This effect was mainly driven by the significant reduction of response times after anodal stimulation (*p* = < .001), as revealed by the post hoc tests for the significant interactions between stimulation and time (χ^2^ = 7.51, df = 2, *p* = .023, *f^2^* = .046). Furthermore, the stimulation, reasoning and time interactions (χ^2^ = 7.75, df = 2, *p* = .021, *f^2^* = .048), the stimulation, argument and time interactions (χ^2^ = 10.93, df = 2, *p* = .004, *f^2^* = .108) and the stimulation, reasoning, argument and time interactions were significant (χ^2^ = 7.62, df = 2, *p* = .022, *f^2^* = .120). After conducting post hoc tests on the four-way interaction, two main results were found (Fig 5B). Firstly, there was a significant shortening in RTs for categorical inductive arguments following anodal stimulation (*p* = < .001) and cathodal stimulation (*p* = .001). Secondly, for categorical inductive arguments, the RTs after stimulation in the anodal (*p* = < .001) and cathodal (*p* = .014) conditions were significantly shorter compared to the sham condition.

*Accuracy rate*. The analysis indicates that the complete model of the ARs has a lower AIC (4564.736 for easy trials and 4306.955 for difficult trials) and Deviance/df (5.277 for easy trials and 5.326 for difficult trials) than the baseline model’s AIC (5209.318 for easy trials and 5071.680 for difficult trials) and Deviance/df (6.683 for easy trials and 7.123 for difficult trials). These findings suggest that the full models are a good fit for the data. Refer to the S4 Table for a detailed breakdown of the full model results.

**Fig 6:**
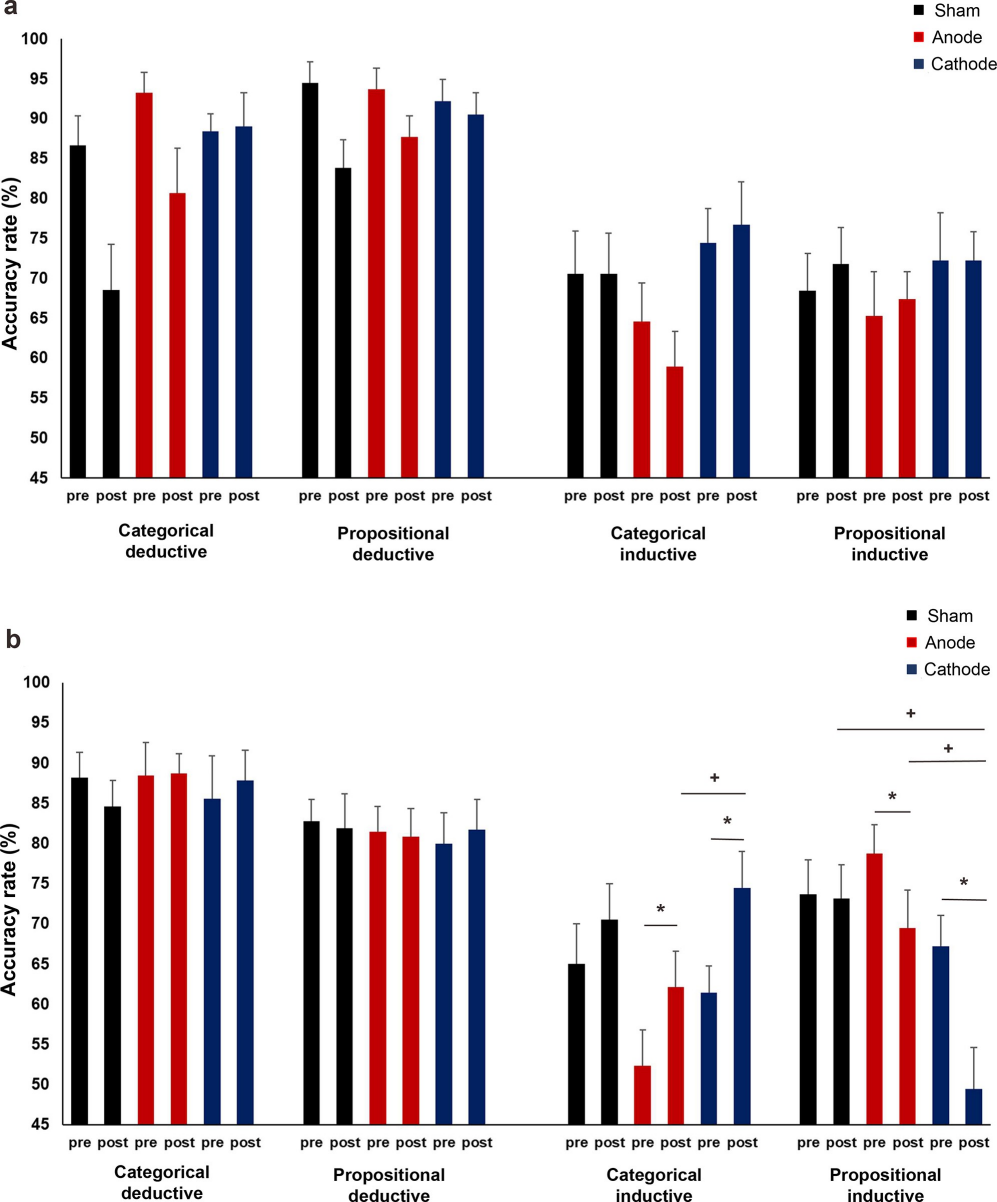
Accuracy rates for deductive and inductive arguments before and after tDCS of the left dorsolateral prefrontal cortex. **(a)** ARs for easy categorical and propositional, deductive and inductive arguments. **(b)** ARs for difficult categorical and propositional, deductive and inductive arguments. ARs for categorical inductive arguments significantly increased after anodal and cathodal stimulation. The post-anodal stimulation ARs for categorical inductive arguments were significantly lower in the anodal than in the cathodal condition. The ARs significantly decreased after anodal and cathodal stimulation for propositional inductive arguments. The post-cathodal stimulation AR was significantly lower than those in the sham and anodal conditions. pre = before stimulation, post = after stimulation, * = indicates significant differences before and after stimulation, + = indicates significant differences in RTs between stimulation conditions. Pairwise comparisons were Bonferroni corrected with a significance level of *p* ≤ 0.05. Data are presented as mean ± standard error of mean.

In the analysis of the easy trials (Fig 6A and S4 Table), it was found that reasoning type (χ^2^ = 470.42, df = 1, *p* = .001, *f^2^* = .322) and argument form (χ^2^ = 15.07, df = 1, *p* = < .001, *f^2^* = .303) had a significant main effect on AR. The participants were more accurate in judging deductive arguments (88.85% ± .69%) than inductive arguments (69.38% ± .58%) and more accurate in judging propositional arguments (80.27% ± .68%) compared to categorical arguments (76.80% ± .59%). The reasoning and argument interaction was also significant (χ^2^ = 12.71, df = 1, *p* = < .001, *f^2^* = .569), with participants having higher accuracy for categorical and propositional deductive arguments compared to their inductive counterparts (all *p*s = < .001). The main effect of time was also significant (χ^2^ = 6.40, df = 1, *p* = .001, *f^2^* = .291), with the overall accuracy decreasing after stimulation (before: 79.65% ± .61%; after: 77.39% ± .66%). Additionally, interactions between reasoning and time (χ^2^ = 10.89, df = 1, *p* = < .001, *f^2^* = .039), argument and time (χ^2^ = 6.57, df = 1, *p* = .010, *f^2^* = .026), and reasoning, argument, and time (χ^2^ = 5.19, df = 1, *p* = .024, *f^2^* = .623) were significant. The post hoc tests for the three-way interactions showed that the decrease in overall accuracy was mainly due to a significant reduction of AR for categorical and propositional deductive arguments (*p*s = < .001) but not categorical and propositional inductive arguments (*p*s = .999). Regarding the specific effects of tDCS, all stimulation-related effects were found to be significant, except for the highest (four-way) interaction (S4 Table). The main effect of the stimulation condition was significant (χ^2^ = 38.96, df = 2, *p* = < .001, *f^2^* = .248), driven by the participant’s higher accuracy in the cathodal condition (82.40% ± .79%) compared to sham (77.36% ± .78%) and anodal (75.93% ± .76%) conditions (*p*s = < .001). The ARs in the sham and anodal conditions were similar (*p* = .546). Post hoc testing for significant stimulation, reasoning, and time interactions (χ^2^ = 6.17, df = 2, *p* = .046, *f^2^* = .677) revealed a significant reduction in accuracy for deductive arguments after sham stimulation (*p* = < .001). Meanwhile, the accuracy for inductive arguments was significantly lower after anodal stimulation compared to sham (*p* = .004). In contrast, the accuracy for deductive arguments was significantly lower after cathodal tDCS compared to sham (*p* = < .001). Furthermore, post hoc tests for stimulation, argument, and time interactions (χ^2^ = 13.79, df = 2, *p* = .001, *f^2^* = .052) showed a significant reduction in accuracy for categorical arguments after sham (*p* = < .001) and anodal (*p* = .039) stimulation. The accuracy for categorical arguments was also higher after cathodal stimulation than sham (*p* = < .001).

The results of the difficult trial analysis (S4 Table) demonstrated that reasoning had a significant main effect on ARs (χ^2^ = 485.27, df = 1, *p* = .001, *f^2^* = .638). Participants were better at accurately judging deductive arguments (85.78% ± .71%) than inductive arguments (65.84% *+* .57%). Further post hoc tests for reasoning and argument interactions (χ^2^ = 56.60, df = 1, *p* = < .001, *f^2^* = .664) revealed that participants were more accurate in judging categorical and propositional deductive arguments than their inductive counterparts (all *p*s = < .001). The interactions between argument and time (χ^2^ = 43.20, df = 1, *p* < .001, *f^2^* = .083), and reasoning, argument, and time were also significant (χ^2^ = 32.44, df = 1, *p* < .001, *f^2^* = .644). Post hoc tests for the three-way interactions revealed a significant increase and decrease in the participants’ accuracy for categorical and propositional inductive arguments after stimulation (all *p*s < .001), respectively. However, there were no significant changes in their accuracy for the deductive arguments. Furthermore, the main and interactive effects of the stimulation were found to be significant, except for its interaction with time (S4 Table). The main effect of the stimulation condition was significant (χ^2^ = 31.26, df = 1, *p* = < .001, *f^2^* = .007) with lower overall accuracy in the anodal (75.33% ± .79%, *p* = .032) and cathodal (72.05% ± .75%, *p* = .032) conditions compared to the sham condition (78.20% ± .81%). The post hoc tests for the highest significant interactions of stimulation, reasoning, argument, and time (χ^2^ = 12.93, df = 2, *p* = .002, *f*^2^ = .786) revealed two findings. First, both anodal and cathodal tDCS significantly increased the accuracy for categorical inductive arguments (anode: *p* = .029, cathode: *p* = .001) and decreased the accuracy for propositional inductive arguments (anode: *p* = .027, cathode: p = < .001) (Fig 6B). Second, for categorical inductive arguments, the accuracy after anodal tDCS was significantly lower than after cathodal tDCS (*p* = < .001) (Fig 6B). Conversely, for propositional inductive arguments, the accuracy after cathodal tDCS was significantly lower than after sham (*p* = < .001) and anodal tDCS (*p* = < .001). No significant differences were found in the accuracy for deductive arguments across stimulation conditions (Fig 6B).

*Summary of tDCS-specific effects*. Stimulating the left DLPFC with anodal and cathodal tDCS led to slower response times and improved accuracy for difficult categorical inductive arguments but decreased accuracy for propositional inductive arguments. On the other hand, anodal and cathodal tDCS did not have a specific effect on the participant’s response times or accuracy for easy inductive arguments, as well as easy and difficult deductive arguments, regardless of argument form.

### Discussion of Experiment 2

The second experiment shows the causal role of the left DLPFC for deductive and inductive reasoning. The results indicated that anodal and cathodal tDCS have specific effects on difficult inductive arguments. Both types of stimulation led to improved reasoning for categorical inductive arguments, as seen through shorter RTs and increased ARs. However, the same stimulations impaired reasoning for propositional inductive arguments, leading to lower ARs. These findings support the hypothesis that the left DLPFC plays a crucial role in inductive reasoning and that tDCS has a more pronounced effect on difficult arguments.

The specific modulation of inductive reasoning by tDCS is supported by imaging studies that consistently show activation of the DLPFC during induction. Verbal syllogistic inductive tasks activate the left DLPFC [14,25,26,61], while figural and numerical inductive tasks activate the bilateral DLPFC [7,76,77]. Left DLPFC activation is related to hypothesis generation, while right DLPFC activation is associated with selecting and implementing previously learned rules during inductive reasoning [78]. Meanwhile, bilateral DLPFC activations for numerical and figural induction are thought to represent the identification of a pattern or rule based on instances that fall under that rule [7,37,76,77]. The late negative event-related potential (ERP) component observed at the PFC during inductive reasoning is also thought to represent the integration of semantic information [79], including those beyond what is presented in the premises but is necessary to assess the likelihood of a conclusion in inductive reasoning. The PFC is likely recruited because it subserves the recall and evaluation of a wide range of world knowledge [26]. This might explain why tDCS does not have a significant effect on deductive reasoning because, although it depends on recognizing and using the logical structure spanning premises and conclusions, deduction relies less on general knowledge [26].

Experiment 2 also showed that tDCS had a specific effect on difficult inductive arguments. It is challenging to explain as the influence of task demands on inductive reasoning has not yet been thoroughly explored using NIBS techniques. Additionally, the induction-related brain activations observed in imaging studies were mostly triggered by non-verbal stimuli [76,80–82]. As previously mentioned, in figural induction tasks, the left DLPFC activation may reflect the process of abstracting and organizing a rule. This could be challenging in difficult cases, as multiple alternative hypotheses must be generated before the correct rule can be induced [76]. Some experts also suggest that DLPFC activation reflects high relational complexity, as seen in the difficult Raven’s Progressive Matrix task, which involves the manipulation of self-generated information [42,80,83]. Goel also proposes that the left PFC reduces uncertainty by processing conceptual connections and logical relations among available information [84]. Therefore, tDCS may affect reasoning for difficult arguments in task-naive participants who rely heavily on the DLPFC due to task complexity and induced uncertainty [80].

Another notable finding from Experiment 2 is enhancing inductive reasoning for categorical arguments after stimulation but not for propositional arguments. This could be because categorical arguments require more background knowledge, resulting in more left DLPFC engagement. For example, the categorical argument "*Cats purr*. *A lion is a cat*. *Therefore*, *lions purr*" requires a deeper understanding of learned biological information (such as animal schemas) than the propositional argument "*If today is Wednesday*, *and tomorrow is Thursday*, *then yesterday was Tuesday*." The present results support this idea, as the stimulation had an effect on RTs and ARs for categorical arguments, but only ARs for propositional arguments. The memory retrieval differences also likely mediated the polarity-independent effects of tDCS. Anodal and cathodal stimulation enhanced judgement for categorical inductive arguments, but impaired judgement for propositional inductive arguments. This was not surprising, as polarity-independent effects on plasticity induction [85–87], working memory and implicit motor learning were reported in humans [88,89]. Studies in rats have shown that the polarity-independent effect is influenced by synaptic input. Stimulation has little effect when synaptic input is too weak, but it is more effective when paired with tasks that induce synaptic plasticity [90]. This supports the argument that performance in categorical inductive reasoning will be facilitated because it increases synaptic activity in the left DLPFC due to a higher memory retrieval process than propositional inductive reasoning.

## General discussion

This study examines the dissociation of neural substrates for deductive and inductive reasoning in the left frontal lobe. In Experiment 1, left IFG anodal tDCS modulated both deduction and induction, while in Experiment 2, left DLPFC anodal and cathodal tDCS only modulated induction. Additionally, tDCS effects were specific for the easy and difficult arguments in Experiments 1 and 2, respectively. Overall, the findings indicate a partial separation of the left frontal lobe regions responsible for the two types of reasoning and demonstrate that stimulation effects depend on argument difficulty. The following section will discuss the task-related findings and provide an interpretation of the main results based on reasoning theories.

Overall, both experiments yielded three task-related findings. First, participants performed better in the deductive than inductive task. This outcome was anticipated as deduction relies on a more accurate and deliberate process than induction [53,91]. Second, the overall performance was better for categorical than propositional arguments, which was also expected because, in terms of logic, categorical arguments are easier to analyze since each component of the premise does not represent a complete claim, whereas, in propositional arguments, each component represents a complete proposition. Moreover, analyzing categorical arguments is more fine-grained since there is a need to investigate and assert a relationship between each class or category. Third, the performance was better for easy than difficult arguments regardless of reasoning type. This suggests efficient categorization of arguments based on difficulty.

Concerning the stimulation-specific effects, the overall results suggest a partial dissociation between deductive and inductive reasoning. Partial mainly because, in Experiment 1, left IFG anodal stimulation impaired both types of reasoning. It is worth noting that in imaging literature, absolute dissociation of task-related brain activations was also not reported; instead, overlapping and co-activated brain regions are frequent findings [7,14,20,25,26]. The dual-process theory can explain the modulation of both reasoning types, which postulates that both heuristic and prior knowledge-based processes (System 1) and analytic processes (System 2) influence deduction and induction, only in different proportions. It is possible that analytic processes follow heuristic processes sequentially or that both processes run in parallel [91]. According to a study by Tsujii et al. (2011), a switching mechanism at the right IFG determines which system dominates depending on the reasoning task. The default system, System 1, is supported by the left IFG through a fronto-parietal pathway, while System 2 is supported by "core" areas like the frontal eye fields and frontopolar prefrontal cortex, as well as "support" areas such as the supramarginal gyrus in the parietal cortex that only engages the left IFG when logical standards are required [33,92,93]. Humans are also thought to use System 2 processes to test conclusions generated by System 1 [24]. Therefore, deduction and induction are unlikely to rely solely on one computational mechanism.

For Experiment 1, the unified computation model by Bara et al. (1999) could explain the results. In this single-process model, deductive and inductive reasoning share common procedures, including construction, integration, conclusion, falsification, and response. Integration and falsification (with two subcomponents: “consistency and equivalence” and the task-specific “search-for-alternatives” component) are strongly shared by deduction and induction [94], and, if modulated by tDCS, can interfere with both reasoning types. The model can also explain the stronger impairment of induction than deduction in Experiment 1, especially concerning the effect of stimulation on the "search-for-alternatives" process. Interference is likely stronger in induction because individuals must search for an alternative model to the one obtained by merging premises and integrating the premises’ model with world knowledge. In contrast, in deduction, individuals only need to search for an alternative model to the one obtained by merging premises [94]. In other words, interference will be more pronounced for reasoning that involves more sub-processes.

In Experiment 2, it was found that left DLPFC stimulation had a specific effect on induction in comparison to Experiment 1. This implies that the brain regions subserving induction and deduction are distinct, validating dual-process theories of reasoning. It is noteworthy that tDCS interfered with inductive reasoning, regardless of polarity. It could be that since induction relies on a rapid heuristic process, any form of stimulation (whether anodal or cathodal) may either enhance or impair it. Identifying which heuristic process is affected by the stimulation can be difficult. It is unclear whether it is the process that taps into the knowledge of generalization and similarity or salient relations (premises and conclusion), or the one that draws on experience and background casual knowledge [95]. In the case of categorical inductive reasoning, it would be beneficial to interrupt or slow down the quick heuristic processes, particularly the knowledge of generalization and similarity or salient relations. This approach is likely to improve task performance because it allows efficient neural computations to infer the properties of the members of a conclusion category based on knowledge about the properties of premises categories [54]. On the other hand, for propositional inductive reasoning, which is impaired by the stimulation, the interference may have enhanced the recollection of irrelevant prior knowledge. Retrieving too much information can lead to the "suppression effect," which can negatively impact the endorsement of conclusions or inferences [24].

Lastly, it was found that argument difficulty had an impact on the stimulation effect. Experiment 1 showed that easy deductive and inductive arguments were specifically affected, suggesting that the left IFG’s role in reasoning may focus more on domain-general processes such as working memory or executive functions and verbal stimulus processing. On the other hand, Experiment 2 revealed that the left DLPFC may be the central node in a larger network of brain regions subserving inductive reasoning, particularly for difficult arguments. The results of Experiment 2 also imply that deductive and inductive judgements rely on different cognitive resources, with argument length-mediated complexity having a greater influence on induction and validity playing a bigger role in deduction [91].

## Limitations

The current study has a few potential limitations that need to be acknowledged. First, the task is based on hypothetical premises, which may not accurately reflect real-world reasoning. Future studies should explore whether tDCS has the same effect on reasoning tasks that simulate real-life events, such as in a virtual reality environment. Second, the study primarily included younger adults. Life experiences can influence our reasoning, so it’s important to be cautious when applying the findings to other age groups. Lastly, the lack of a control or parallel task is a significant limitation because it makes it hard to distinguish the specific effects of tDCS on reasoning from other cognitive processes like memory, attention, perception, and decision-making.

## Conclusions

This study provides evidence that brain areas responsible for deduction and induction (left IFG) and induction alone (left DLPFC) can be partially dissociated, supporting dual-process theories of reasoning. Understanding these neural mechanisms in the left frontal lobe can be valuable, especially for older adults who heavily rely on prior knowledge and experience to solve everyday problems [96]. Targeting the left DLPFC through brain stimulation may help older adults address difficulties related to integrating various relationships when problem-solving [97].

## Supporting information

S1 Table The participants’ and experimenter’s correct guesses and the results of the chi-square tests. (DOCX)

S2 Table Results of the GLM analysis of the reaction times (RTs) and accuracy rates (ARs) in Experiment 1. (DOCX)

S3 Table Results of the GLM analysis of the reaction times (RTs) in Experiment 2. (DOCX)

S4 Table Results of the GLM analysis of the accuracy rates (ARs) in Experiment 2. (DOCX)

S1 Data Data Experiment 1 RT and AR. (XLSX)

S2 Data Data Experiment 2 RT and AR. (XLSX)
