# Supplementary material for: Partially dissociative role of the left inferior frontal gyrus and left dorsolateral prefrontal cortex in reasoning
Source: PLoS One. 2024 Dec 2;19(12):e0312919. doi: 10.1371/journal.pone.0312919 (PMC11611129; doi:10.1371/journal.pone.0312919)
Supplement: S1 Table — (DOCX) [file pone.0312919.s001.docx]

**S1 Table.** The participants’ and experimenter’s correct guesses and the results of the chi-square tests.

| *Experiment 1* | | | | | | | |
| --- | --- | --- | --- | --- | --- | --- | --- |
| Participants | | | | Experimenter | | | |
| Stimulation condition | Correct Guesses | X² | *p* | Stimulation condition | Correct Guesses | X² | *p* |
| Sham | 4/20 (20%) | 1.19 | .551 | Sham | 6/20 (30%) | 0.43 | .803 |
| Anode | 7/20 (35%) |  |  | Anode | 7/20 (35%) |  |  |
| Cathode | 5/20 (25%) |  |  | Cathode | 8/20 (40%) |  |  |
| *Experiment 2* | | | | | | | |
| Participants | | |  | Experimenter | | | |
| Stimulation condition | Correct Guesses | X² | *p* | Stimulation condition | Correct Guesses | X² | *p* |
| Sham | 6/19 (31.58%) | 2.75 | .313 | Sham | 10/19 (52.63%) | 5.58 | .140 |
| Anode | 4/19 (21.05%) |  |  | Anode | 6/19 (31.58%) |  |  |
| Cathode | 8/18 (44.44%) |  |  | Cathode | 4/18 (22.22%) |  |  |
