# Supplementary material for: Partially dissociative role of the left inferior frontal gyrus and left dorsolateral prefrontal cortex in reasoning
Source: PLoS One. 2024 Dec 2;19(12):e0312919. doi: 10.1371/journal.pone.0312919 (PMC11611129; doi:10.1371/journal.pone.0312919)
Supplement: S2 Table — (DOCX) [file pone.0312919.s002.docx]

**S2 Table.** Results of the GLM analysis of the reaction times (RTs) and accuracy rates (ARs) in Experiment 1.

| **RTs** | | | | |
| --- | --- | --- | --- | --- |
|  | Wald χ² | df | *p-*value | Cohen’s *f*² |
| *Easy trials* |  |  |  |  |
| Stimulation | 34.89 | 2 | <.001* | .005 |
| Reasoning | 26.89 | 2 | <.001* | .039 |
| Time | 22.05 | 1 | <.001* | .041 |
| Stimulation x reasoning | 2.16 | 4 | .706 | .076 |
| Stimulation x time | 15.76 | 2 | <.001* | .089 |
| Reasoning x time | 26.54 | 2 | <.001* | .056 |
| Stimulation x reasoning x time | 14.19 | 4 | .007* | .184 |
| *Difficult trials* |  |  |  |  |
| Stimulation | 3.29 | 2 | .193 | .023 |
| Reasoning | 78.31 | 2 | <.001* | .049 |
| Time | 4.86 | 1 | .027* | .028 |
| Stimulation x reasoning | 9.16 | 4 | .057 | .097 |
| Stimulation x time | 3.43 | 2 | .180 | .020 |
| Reasoning x time | 12.73 | 2 | .002* | .092 |
| Stimulation x reasoning x time | 2.10 | 4 | .717 | .140 |
| **ARs** | | | | |
|  | Wald χ² | df | *p*-value | Cohen’s *f²* |
| *Easy trials* |  |  |  |  |
| Stimulation | 9.75 | 2 | .008* | .182 |
| Reasoning | 249.34 | 2 | <.001* | .581 |
| Time | .96 | 1 | .326 | .075 |
| Stimulation x reasoning | 9.88 | 4 | .042* | .661 |
| Stimulation x time | 7.46 | 2 | .024* | .006 |
| Reasoning x time | 6.88 | 2 | .032* | .620 |
| Stimulation x reasoning x time | 21.29 | 4 | <.001* | .701 |
| *Difficult trials* |  |  |  |  |
| Stimulation | 10.09 | 2 | .006* | .012 |
| Reasoning | 186.42 | 2 | <.001* | .421 |
| Time | .03 | 1 | .859 | .102 |
| Stimulation x reasoning | 21.37 | 4 | <.001* | .535 |
| Stimulation x time | 13.41 | 2 | .001* | .031 |
| Reasoning x time | 40.54 | 2 | <.001* | .445 |
| Stimulation x reasoning x time | 22.57 | 4 | <.001* | .560 |

* = indicate significant results (*p* < 0.05), df = Degrees of freedom, Wald χ² = Wald chi-square test.
