# Supplementary material for: Partially dissociative role of the left inferior frontal gyrus and left dorsolateral prefrontal cortex in reasoning
Source: PLoS One. 2024 Dec 2;19(12):e0312919. doi: 10.1371/journal.pone.0312919 (PMC11611129; doi:10.1371/journal.pone.0312919)
Supplement: S3 Table — (DOCX) [file pone.0312919.s003.docx]

**S3 Table.** Results of the GLM analysis of the reaction times (RTs) in Experiment 2.

|  | Wald χ² | df | *p-*value | Cohen’s *f*² |
| --- | --- | --- | --- | --- |
| *Easy trials* |  |  |  |  |
| Stimulation | 11.54 | 2 | .003* | .081 |
| Reasoning | 13.95 | 1 | <.001* | .106 |
| Argument | 67.72 | 1 | <.001* | .035 |
| Time | .16 | 1 | .690 | .014 |
| Stimulation x reasoning | 4.55 | 2 | .103 | .033 |
| Stimulation x argument | 7.55 | 2 | .023* | .145 |
| Stimulation x time | 6.07 | 2 | .048* | .011 |
| Reasoning x argument | .68 | 1 | .410 | .016 |
| Reasoning x time | 10.96 | 1 | <.001* | .023 |
| Argument x time | 1.40 | 1 | .238 | .125 |
| Stimulation x reasoning x argument | 6.48 | 2 | .039* | .212 |
| Stimulation x reasoning x time | 1.48 | 2 | .477 | .034 |
| Stimulation x argument x time | .75 | 2 | .687 | .150 |
| Reasoning x argument x time | .13 | 1 | .718 | .176 |
| Stimulation x reasoning x argument x time | 4.11 | 2 | .128 | .252 |
| *Difficult trials* |  |  |  |  |
| Stimulation | 21.86 | 2 | <.001* | .065 |
| Reasoning | 1.01 | 1 | .315 | .072 |
| Argument | 6.58 | 1 | .010* | .012 |
| Time | 18.45 | 1 | <.001* | .017 |
| Stimulation x reasoning | 1.20 | 2 | .548 | .003 |
| Stimulation x argument | 4.99 | 2 | .082 | .017 |
| Stimulation x time | 7.51 | 2 | .023* | .046 |
| Reasoning x argument | 2.86 | 1 | .091 | .017 |
| Reasoning x time | 4.60 | 1 | .032* | .046 |
| Argument x time | 9.98 | 1 | .002* | .097 |
| Stimulation x reasoning x argument | 4.35 | 2 | .113 | 019 |
| Stimulation x reasoning x time | 7.75 | 2 | .021* | .048 |
| Stimulation x argument x time | 10.93 | 2 | .004* | .108 |
| Reasoning x argument x time | .91 | 1 | .339 | .100 |
| Stimulation x reasoning x argument x time | 7.62 | 2 | .022* | .120 |

* = indicate significant results (*p* < 0.05), df = Degrees of freedom, Wald χ² = Wald chi-square test.
