# Supplementary material for: Partially dissociative role of the left inferior frontal gyrus and left dorsolateral prefrontal cortex in reasoning
Source: PLoS One. 2024 Dec 2;19(12):e0312919. doi: 10.1371/journal.pone.0312919 (PMC11611129; doi:10.1371/journal.pone.0312919)
Supplement: S4 Table — (DOCX) [file pone.0312919.s004.docx]

**S4 Table.** Results of the GLM analysis of the accuracy rates (ARs) in Experiment 2.

|  | Wald χ² | df | *p-*value | Cohen’s *f*² |
| --- | --- | --- | --- | --- |
| *Easy trials* |  |  |  |  |
| Stimulation | 38.96 | 2 | <.001* | .248 |
| Reasoning | 470.42 | 1 | .001* | .322 |
| Argument | 15.07 | 1 | <.001* | .303 |
| Time | 6.40 | 1 | .001* | .219 |
| Stimulation x reasoning | 40.63 | 2 | <.001 | .609 |
| Stimulation x argument | 12.85 | 2 | .002* | .035 |
| Stimulation x time | 10.83 | 2 | .004* | .039 |
| Reasoning x argument | 12.71 | 1 | <.001* | .569 |
| Reasoning x time | 10.89 | 1 | <.001* | .577 |
| Argument x time | 6.57 | 1 | .010* | .026 |
| Stimulation x reasoning x argument | 22.06 | 2 | <.001* | .665 |
| Stimulation x reasoning x time | 6.17 | 2 | .046* | .677 |
| Stimulation x argument x time | 13.79 | 2 | .001* | .052 |
| Reasoning x argument x time | 5.19 | 1 | .024* | .623 |
| Stimulation x reasoning x argument x time | .57 | 2 | .751 | .082 |
| *Difficult trials* |  |  |  |  |
| Stimulation | 31.26 | 2 | <.001* | .007 |
| Reasoning | 485.27 | 1 | .001* | .638 |
| Argument | 1.83 | 1 | .176 | .129 |
| Time | .03 | 1 | .856 | .134 |
| Stimulation x reasoning | 10.31 | 2 | .006* | .780 |
| Stimulation x argument | 60.62 | 2 | <.001* | .013 |
| Stimulation x time | 1.75 | 2 | .417 | .012 |
| Reasoning x argument | 52.60 | 1 | <.001* | .664 |
| Reasoning x time | .11 | 1 | .736 | .064 |
| Argument x time | 43.20 | 1 | <.001* | .083 |
| Stimulation x reasoning x argument | 88.49 | 2 | .001* | .786 |
| Stimulation x reasoning x time | 8.29 | 2 | .016* | .780 |
| Stimulation x argument x time | 17.49 | 2 | <.001* | .013 |
| Reasoning x argument x time | 32.44 | 1 | <.001* | .644 |
| Stimulation x reasoning x argument x time | 12.93 | 2 | .002 | .786 |

* = indicate significant results (*p* < 0.05), df = Degrees of freedom, Wald χ² = Wald chi-square test.
